# Morphological characteristics of preparator air-scribe marks: Implications for taphonomic research

**DOI:** 10.1371/journal.pone.0209330

**Published:** 2018-12-20

**Authors:** Logan A. Wiest, Joseph V. Ferraro, Katie M. Binetti, Steven L. Forman, Donald A. Esker, Mzalendo Kibunjia, Jean-Philip Brugal, Bernd Zechmann

**Affiliations:** 1 Department of Geosciences, and Institute of Archaeology, Baylor University, Waco, Texas, United States of America; 2 Department of Anthropology, and Institute of Archaeology, Baylor University, Waco, Texas, United States of America; 3 National Museums of Kenya, Nairobi, Kenya; 4 Aix Marseille University, CNRS, Ministère de la Culture, UMR 7269 LAMPEA, Aix-en-Provence, France; 5 Center for Microscopy and Imaging, Baylor University, Waco, Texas, United States of America; Ecole Normale Supérieure de Lyon, FRANCE

## Abstract

Taphonomic analyses of bone-surface modifications can provide key insights into past biotic involvement with animal remains, as well as elucidate the context(s) of other biostratinomic (pre-burial) processes, diagenesis, excavation, preparation and storage. Such analyses, however, first require researchers to rigorously disambiguate between continuums of damage morphologies prior to attributing individual marks to specific actors and effectors (e.g., carnivore tooth, stone tool cutting edge, etc.). To date, a number of bone-modifying agents have been identified, and criteria for identifying their traces have been published. Relatively little research, however, has focused on bone-surface modifications imparted during specimen preparation. Herein we report that air scribes, small pneumatic tools commonly used for preparation in museum contexts, can generate unintentional marks that may mimic surficial modification caused by carnivores. To aid investigators in assessing the hypothesis that a mark in question is derived from air-scribe preparation activities, we provide high-resolution, detailed morphological information imaged with scanning electron microscopy (SEM). The main diagnostic characteristic of air-scribe damage is the occurrence of sequential, variously spaced, sub-millimeter scallop-like stepped bone removals. This morphology can resemble damage imparted by carnivore teeth. In contrast to marks produced by trampling, stone tools and carnivores, however, no continuous internal features, such as linear microstriations, were observed within grooves produced by the air scribe. Thus, the presence of such features can be used to disprove an air-scribe origin. A culmination of the morphological criteria presented herein, cross-cutting relationships with other surficial features (e.g., diagenetic discoloration, weathering textures), the position of occurrence, and an overall contextual framework for the assemblage is suggested for accurate identification of such traces. The ability to recognize or disprove air-scribe damage will allow researchers to confidently proceed with interpreting past biological and sedimentological interactions with animal remains.

## Introduction

Taphonomic analyses of bone-surface modifications can provide key insights into past biotic involvement with animal remains [1–12]. Such studies have proven invaluable in elucidating the diets and paleoecologies of insects, dinosaurs, mammalian carnivores, and Paleolithic hominins, among others [13–24]. These analyses, however, first require researchers to rigorously disambiguate between continuums of damage morphologies prior to attributing individual marks to specific agents and effectors (e.g., carnivore tooth, stone tool cutting edge, etc.). The recent discovery that crocodiles can produce linear damage morphologies that may mimic stone-tool marks highlights how descriptions and explanations of previously unexplored bone-surface modifications can dramatically alter interpretations of ancient assemblages [5, 6, 12, 24, 25].

In recent years, an increasing number of bone-modifying agents have been identified, and criteria for identifying their marks have been published ([26] and refs therein; [27]). Most of these studies focus on biostratinomic (pre-burial) agents. However, the taphonomic history of a bone or fossil (hereafter, bone) spans from the death of the animal until the present day [27–29]. Taphonomic analyses typically occur after bones have been excavated, cleaned, and archived. Relatively little research has focused on bone-surface modifications imparted during this latter portion of the taphonomic record (though see [27] and references therein). This is potentially problematic because excavation and preparation activities can create additional marks on bone surfaces [30, 31]. In some instances, identifying this modern damage is relatively straightforward. Such is the case when cross-cutting relationships can be applied in relation to other taphonomic or diagenetic features preserved on the bone (e.g., staining, root etching, weathering texture) to deduce the relative timing of the trace (i.e., bone-surface mark) generation [32–34]. Other occurrences, especially subtle and/or isolated traces, require further analysis prior to inferring agency, effector, and timing [27, 30].

In this paper we investigate bone-surface modifications experimentally imparted by an air scribe, a small pneumatic tool commonly used in museum contexts. These hand-held tools utilize compressed air in conjunction with an internal piston to drive a carbide stylus in a high-frequency, reciprocating movement [35]. This action is then applied by the operator to dislodge adhering matrix during preparation. Despite the widespread use of air scribes and obvious potential for imparting traces on specimens, diagnostic criteria for identifying air-scribe damage is currently absent from the paleontological, ichnological, archaeological, and taphonomic literatures. The aim of the present study is to provide morphological information to aid investigators in assessing the hypothesis that a bone-surface feature in question was derived from air-scribe preparation.

## Materials and methods

The experimental air-scribe-induced features were generated by a Chicago Pneumatic Air Scribe (CP9361) with an approximately 0.5 mm wide, pointed carbide tip (see [35]) on 26 non-diagnostic Pleistocene chelonian carapace fragments and mammalian bone remains from the Chalbi Basin, northern Kenya, which were exported for destructive geochemical analyses [36, 37]. All specimens possessed well-preserved cortical-bone surfaces. A variety of reasonable transverse speeds, approach angles, and downward pressures were applied to the air scribe by a single operator with the objective of introducing a morphologically diverse range of markings. Each fragment was modified by the air scribe multiple times (typically n > 10). Specimen hardness is between 4 and 5 on the Mohs hardness scale as determined by scratching the fossils with select minerals of known hardness.

The carnivore-induced modifications analyzed in this study were generated by providing 10 feral pig (*Sus scrofa*) and domestic bovine (*Bos taurus*) long bones to domestic canines (*Canis lupus familiaris*). The resulting bone-surface modifications ranged from a few isolated occurrences to an abundance of overlapping traces. Fresh bone hardness is determined to be between 3 and 4 on Mohs hardness scale.

Experimental lithic-tool-induced marks were generated using basalt flakes, hammerstones, and anvils during ongoing butchery experiments with domestic goats (*Capra aegagrus hircus*). A total of 21 modified long bone fragments were analyzed in this study. Specimens are between 3 and 4 on Mohs hardness scale.

**Fig 1:**
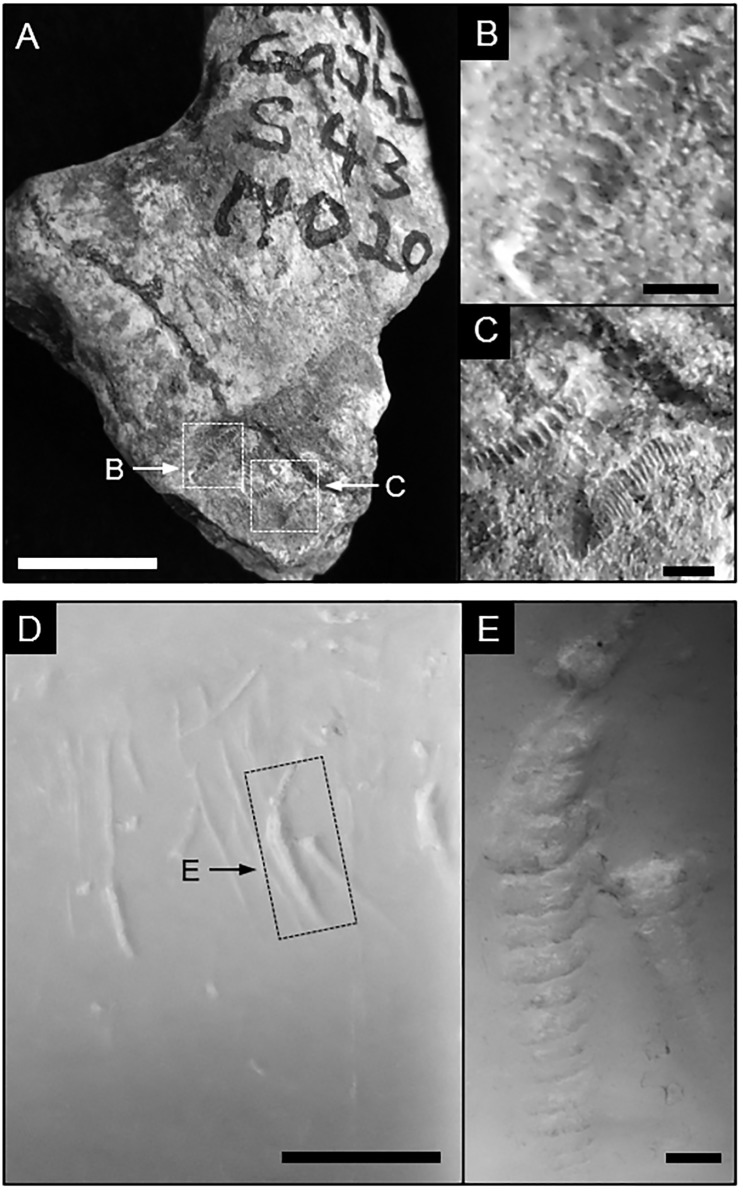
Archived bone fragment (Sample ID—LA91-S43#020) imaged with a digital camera. Sample was recovered from the Lokalalei 1 archaeological site, West Turkana, Kenya, and contains unintentionally induced markings from air-scribe preparation techniques. A) scale bar = 1.0 cm; B, C) scale bars = 1.0 mm. Notice the similarities between these marks and the carnivore-induced tooth scores in Fig 1D and E. D) Carnivore-induced tooth scores and pits on a bovid long bone (Sample 4–2) under a stereomicroscope; scale bar = 1.0 cm. E) Close-up of area highlighted in D; scale bar = 1.0 mm.

The museum specimen depicted in Fig 1A–1C contains unintentionally produced air-scribe damage on a bone fragment (ca. 2.4 My; Sample LA91-S43#020) recovered from the Lokalalei 1 archaeological site, West Turkana, Kenya, and is housed in the National Museums of Kenya [38].

Macroscopic bone surface modifications were examined and imaged under low-angle, polarized-LED illumination with limited background light. Visual inspection was conducted with the extended depth of field (EDOF) setting on a Dino-Lite Edge digital microscope (AM7915 Series) with DinoCapture 2.0 software (v. 1.5.26.C), and a stereo-microscope (SZX16, Olympus Corp., Tokyo, Japan). Scanning Electron Microscopy (SEM) images were taken of uncoated samples at low vacuum conditions either with a FEI Versa 3D scanning electron microscope (FEI, Hillsboro, OR, USA) or a Hitachi TM3030 Plus tabletop scanning electron microscope (Hitachi, Tokyo, Japan). All micrographs were taken with 15kV and at ~10mm working distance at the Center for Microscopy and Imaging at Baylor University (Waco, Texas, U.S.A).

## Results

### Air-scribe marks

**Fig 2:**
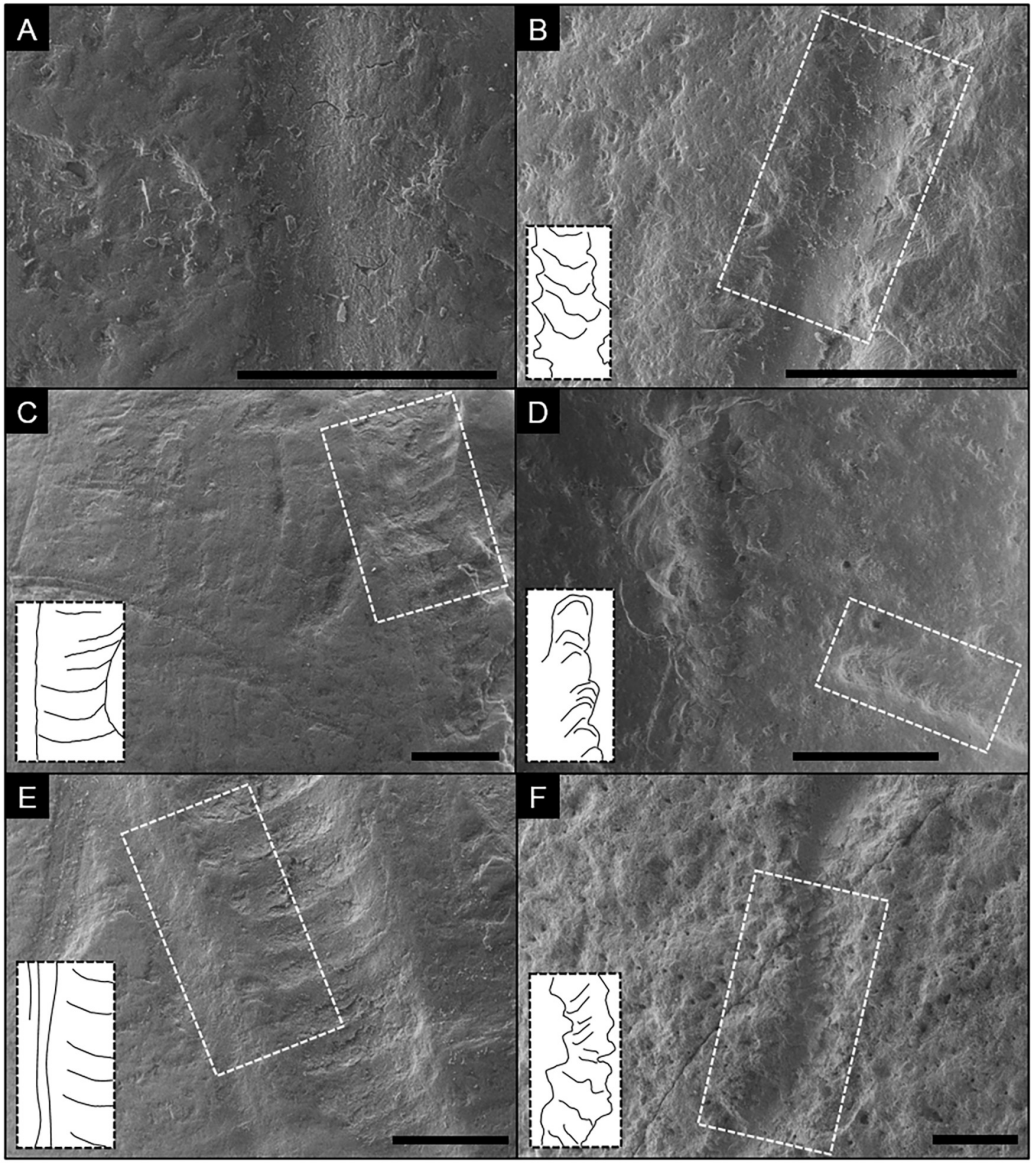
Comparison of carnivore-induced tooth scores (left column) and air-scribe-induced grooves (right column) under SEM. Insets are interpretive drawings of highlighted areas. A) Segment of a smooth tooth score with relatively smooth margins and faint, internal grooves exhibiting continuity on a bovid long bone; scale bar = 0.5 mm. B) Segment of a transition within an air-scribe groove from a chatter-like morphology (upper half) into a smooth-based groove on a fossilized bone fragment; scale bar = 1.0 mm; Notice the rougher margins in comparison to A. C) Shallow tooth score with chatter marks, a smooth left margin, and an irregular right margin on a bovid long bone; scale bar = 1.0 mm. D) shallow (lower left) and deep (top to bottom) air-scribe grooves on a fossilized bone fragment; scale bar = 1.0 mm. Notice the similarities between the chatter morphology of the lower-left mark and the scores depicted in C and E. Also note the relatively rougher margins. E) Tooth score with pronounced chatter markings and a long-axis-parallel, smooth left shoulder with through-going continuity on a bovid long bone; scale bar = 1.0 mm. F) sinuous air-scribe groove with faint chatter marks, rough margins, and an absence of through-going continuity on a fragment of a chelonian carapace; scale bar = 1.0 mm.

**Fig 3:**
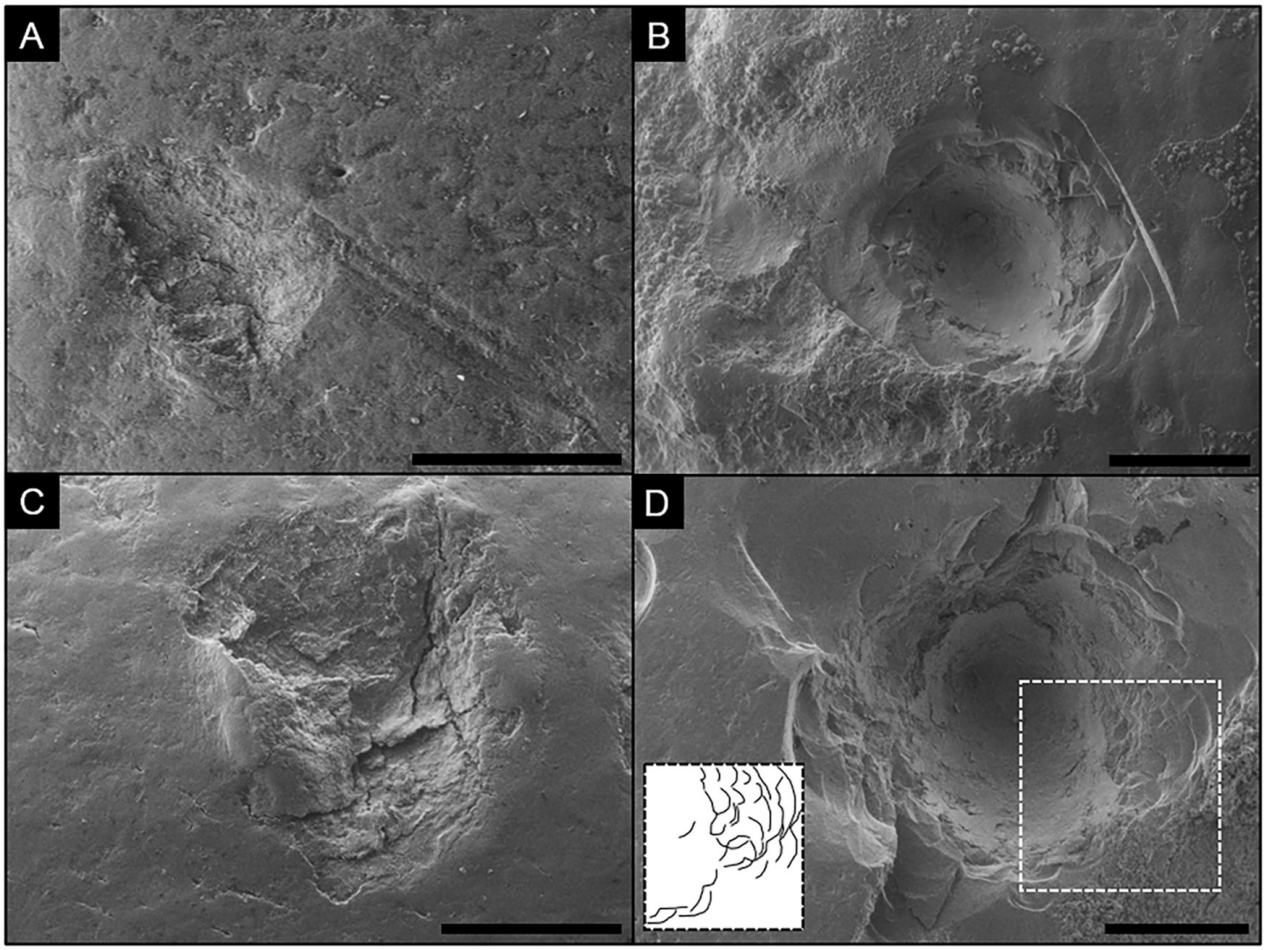
Comparison of carnivore-induced tooth pits (left column) and air-scribe-induced pits (right column) under SEM. Notice the rosette-like morphology of the pits depicted on the right. A) Shallow tooth pit with associated microgrooves (faint score) on a bovid long bone; scale bar = 1.0 mm. B) Shallow pit generated by an air-scribe on a fossilized mammalian bone fragment. Notice the sequential, stepwise scalloping that creates flat benches on the various layers of cortical lamellae; scale bar = 1.0 mm. C) tooth pit with raised margins on the left and right sides; scale bar = 1.0 mm. D) Somewhat deeper air-scribe-induced pit (relative to B) on a mammalian fragment; scale bar = 1.0 mm. Inset is interpretive drawing of highlighted area.

The experimental air-scribe marks are categorized in two classes: grooves (Figs 1A and 2: right column) and pits (Fig 3: right column; see [39] for definitions of general morphological terms). Air-scribe pits vary in diameter and depth (~ mm scale), which is dependent on the pressure and duration of the application of the chisel tip to the material. The pits share a common, ‘rosette’-like morphology despite the amount or duration of applied pressure by the operator. This rosette appearance is caused by the repetitive removal of arcuate flakes that leave behind sub-millimeter scallops (Fig 3B and 3D). The individual scallops often have a flat base that cleaves along cortical lamellae boundaries. Scallops toward the center of the pit penetrate deeper bone layers, generating a crater-like overall morphology with a rosette-like crater wall (Fig 3B and 3D).

Air-scribe grooves, similar to the air-scribe pits, also contain rough outer margins composed of variably-shaped, sub-millimeter, negative flake scars that result from the high-frequency reciprocating impacts of the bit (Fig 2B, 2D and 2F). The gross morphology of the grooves (e.g., depth, sinuosity, etc.) varies dependent on the pressure and direction applied by the operator and is undiagnostic. The basal floor of the grooves varies from smooth (Fig 2B) to scalloped and rough (Fig 2D). ‘Chatter-marked’ refers to the sequential arcuate ridges that are perpendicular to the long axis of the groove. Small-scale features that exhibit linear continuity (e.g., shouldering, microgrooves, etc.) within or associated with the air-scribe grooves were not observed for the samples despite the amount or duration of applied pressure by the operator.

#### Comparison to linear marks made by carnivore teeth

Scores made by carnivore teeth (linear grooves) and air-scribe grooves overlap in size (~ 1–2 mm) and depth (typically < 1 mm; Fig 1B). Traces produced by either mechanism can have a smooth internal floor (Fig 2A and 2B), or a rough base with fine ridges perpendicular to the long axis (Fig 2C–2F). Unlike the marks produced by carnivores, grooves produced by an air scribe have a sequential scalloping/cratering effect that generates an irregular outer margin (Fig 2B, 2D and 2F). Air-scribe-induced damage does not exhibit any features that are continuous across the series of scallops, such as microgrooves, microstriations, or raised shoulders (cf. Fig 2A and 2E; [34]).

#### Comparison to pits made by carnivore teeth

Pit-like features created by air scribes overlap in gross morphology and size with pits generated by carnivore teeth (Fig 3). Both exhibit a sub-circular shape with rough outer margins (Fig 3, cf. left and right columns; [40, 41]), and both can crosscut multiple layers of cortical lamellae and result in the removal of surficial cortical bone (Fig 3). Pits generated by air scribes have a ‘rosette’-like morphology that is generated as a series of steps that increase in depth toward the center of the trace (Fig 3B and 3D). Pits generated by carnivore teeth, on the other hand, do not exhibit the rosette morphology or sequential scalloping, but instead, are more irregularly shaped and have a crushed and flattened base (Fig 3A and 3C; [26]). Tooth pits in this study also have a relatively less-steep sidewall, and occasionally exhibit a raised margin (Fig 3C), whereas the air-scribe marks are typically steeper-walled and do not possess raised, shoulder-like margins (Fig 3B and 3D).

#### Comparison to lithic cut marks and sedimentary abrasion

The air-scribe grooves are distinct from cut marks produced by lithic technology [14, 42, 43]. Specifically, microstriations and/or associated scratches were not observed with any of the traces produced by the air scribe [42, 26]. The sequential, arcuate scallops, which are ubiquitous with the air-scribe marks, are not observed in the linear lithic marks. Air-scribe damage also has a relatively rougher outer margin, which lacks any raised shoulders (Fig 2B, 2D and 2F).

**Fig 4:**
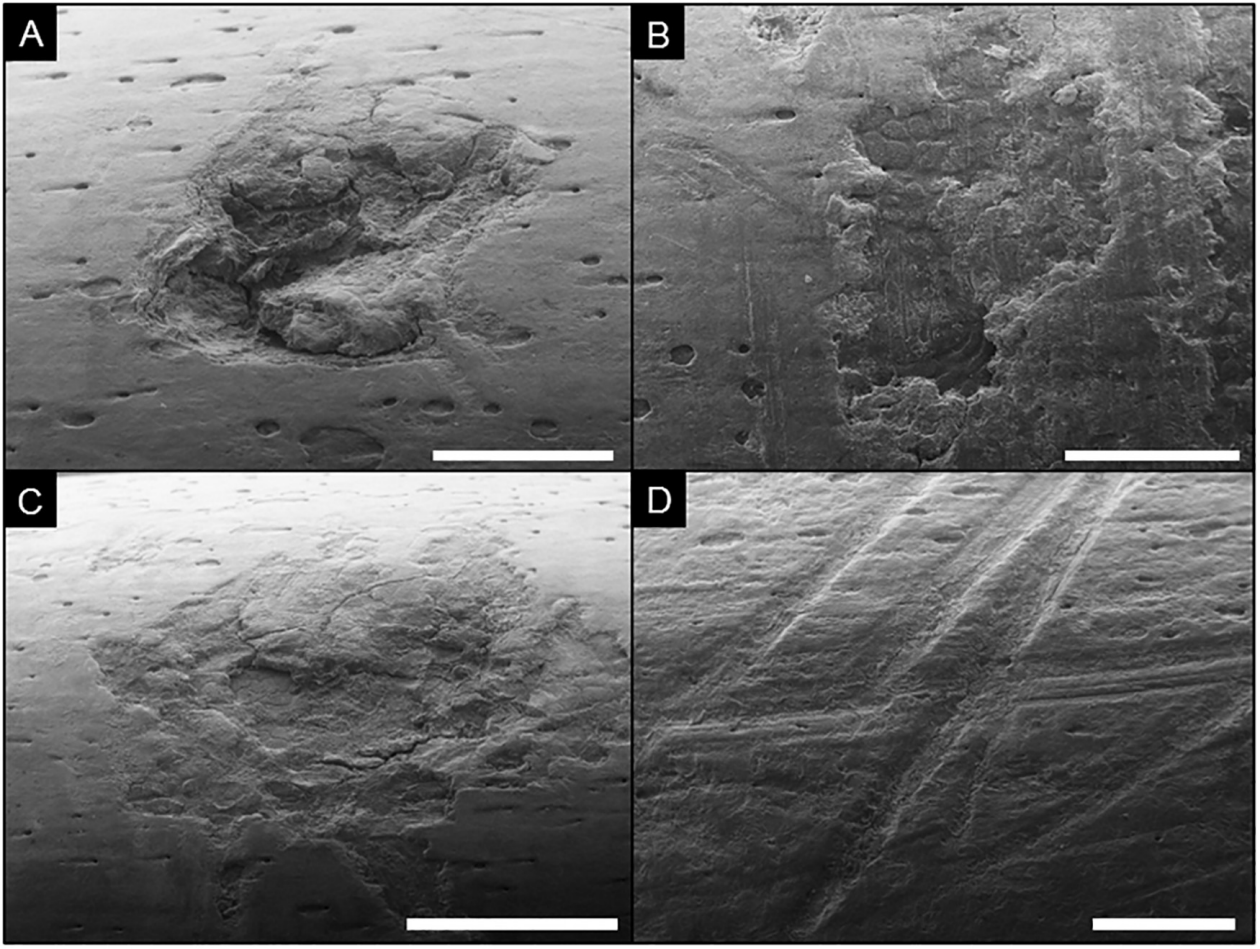
Experimental lithic-tool damage on goat bones under SEM. A) Percussion pit; notice the roughness and cracking that is not apparent on the air-scribe damage depicted in Fig 3B and 3D; scale bar = 2 mm. B) Irregularly shaped percussion pit with abundant (vertically oriented) microstriations; scale bar = 2 mm. C) Percussion pit with associated microstriations and fine-scale fractures; scale bar = 1 mm. D) Multiple, cross-cutting cut marks with internal microstriations and smooth margins; scale bar = 2 mm.

Likewise, air-scribe grooves are distinct from sedimentary abrasion. Striations caused by trampling are generally fine and shallow, regardless of the particle size of the substrate, and can create a polish on the bone surface [43]. Whereas air-scribe grooves can also be shallow, they are relatively wide in comparison to sedimentary abrasion (Fig 4B). Furthermore, surficial polish was not observed with the experimental air-scribe damage. Chatter marks, such as those depicted in Fig 1, were not observed in sedimentary abrasion marks produced by experimental trampling [43].

#### Comparison to lithic percussion pits

The gross size and shape of air-scribe pits can somewhat resemble hammerstone percussion pits (compare Figs 3B and 4C), but the latter differ from air-scribe pits and grooves by having internal and associated microstriae, which are not observed in air-scribe marks (Fig 4; [2]). In addition, sequential, arcuate scallops are unique to the air-scribe marks and are not observed in percussion pits.

## Discussion

Morphological descriptions of air-scribe marks provide researchers with a means to potentially identify or falsify post-excavation damage that confounds bone-surface modification studies. This has several important implications, as well as raises several questions.

### Explanatory mechanisms

Morphological differences between air-scribe-induced marks and alternative forms of bone modification can be attributed to the different mechanics responsible for the removal of material. Namely, carnivore teeth and non-mechanical tools, with the exception of percussion, introduce a relatively slow, static load to the bone surface. Alternatively, the high-frequency reciprocating impacts of the air-scribe bit cause repetitive dynamic loading. Static vs. dynamic loading dictates the shape of the expelled particles (flakes), and thus influences the morphology of the resulting trace [44].

### SEM

The key criteria for differentiating air-scribe marks (e.g., small-scale scallops, rough outer edge, etc.) may be observed with a hand lens (10x) or low-power microscopy (~10-40x), and identification does not necessarily require SEM. However, the SEM yields an effective method for imaging specifically the morphology of these traces [45–47]. Glares, color variations, and limited depths of focus, which can introduce artifacts to the images, are the main limitations when light microscopy is utilized. These complications were eliminated when SEM was used to evaluate the ultrastructure of air-scribe damage on bones. Furthermore, modern SEMs do not require coating of non-conductive samples, such as bones, with a fine layer of conductive metal (e.g. iridium, gold, palladium) or carbon, which can induce artifacts as well [48, 49]. In low vacuum/variable pressure mode, as applied in this study, non-conductive samples, such as bones, can be imaged in the SEM in their native state [50–53]. However, the SEM does have limitations. The vacuum chambers of the SEMs have a finite size and limit the size of the sample that can be observed. Of the two SEMs used, the FEI Versa 3D SEM has the largest vacuum chamber and is capable of imaging bone fragments up to approximately 20 cm in length. The Hitachi TM3030 Plus tabletop SEM is limited to samples < 10 cm in length, in which case microscopy needs to be focused on the center region of the sample. Cutting the sample can circumvent SEM-based sample length limitations for studies if sample modification is acceptable. Often cutting the sample is ill advised or not practical for rare finds, in which case making a mold of the structure in question could allow one to still utilize an SEM approach [54].

### Comparison with damage from other tools

Table 1 provides a list of observed characteristics for comparing air-scribe damage with marks produced by various other tools used in excavation and preparation; adapted from [27]. Based on these characteristics, the air-scribe damage is most similar to marks produced by a screwdriver and an awl but exhibits several important differences (Table 1). The screwdriver and awl both produce microstriations during a friction-inducing movement, whereas air-scribe damage does not produce microstriations. Screwdrivers and awls do not produce internal flakes during an impact movement, whereas the air scribe does. The latter is attributed to the high-frequency reciprocating movement that is unique to the air scribe. The air scribe is also distinct from the other tools listed in Table 1 in the sense that both friction and impact motions of the air scribe do not produce different characteristics beyond the gross morphology (e.g., pits versus grooves).

**Table 1:** Characteristics of bone damage produced by various tools; adapted from [27]. I = Impact; F = Friction; L = Linear; S = Sub-rounded; X = present; - = absent.

Tools		Movement	Shape	Cross -section	Lateral side		Wall		
		I/F	Linear/Sub-rounded	U/V	Hertzian cones	Detachments	Micro-striations	Internal flakes	Micro-cracks
Metallic	1. Small pick^a^	I	L	V	-	X	X	X	X
	2. Chisel+Hammer^a^	I	L	V	-	X	X	-	X
	3. Trowel^a^	I	S	U	-	X	X	X	X
		F	L	V	X	X	X	X	X
	4. Screwdriver^a^	I	S	U	-	X	-	-	X
		F	L	U	-	X	X	X	X
	5. Awl^a^	I	S	U	-	-	-	-	X
		F	L	U	X	X	X	X	X
	6. Scalpel^a^	I	S	V	-	X	-	X	X
		F	L	V	X	X	X	X	X
	7. Palette knife^a^	I	S	V	-	-	-	X	X
		F	L	V	X	X	X	X	X
	8. Flat-tip probe^a^	I	S	U	-	X	-	X	X
		F	L	U	X	X	X	X	X
	9. Fine-tip probe^a^	I	S	U	-	X	-	X	X
		F	L	V	X	X	X	X	X
	10. Air scribe^b^	I	S	U	X	X	-	X	X
		F	L	U	X	X	-	X	X
Wood	11. Skewer^a^	F	-	-	-	-	-	-	-
Plastic	12. Surgical pick^a^	F	L	-	-	-	-	-	-

^a^ Data from [27];

^b^ Data from this study.

### Analytical issues

Differences in fossilization and preservation that affect the physical properties of a bone can potentially influence the morphology of the resulting trace. The experimental carnivore-induced and lithic-tool-induced traces were generated on fresh bone containing collagen (Figs 1B and 4, respectively), whereas the unintentional and experimental air-scribe marks (Figs 1A and 2: right columns, and 3: right column) were produced on fully fossilized material. This difference in preservation inherently influences the physical properties of the material (e.g. hardness, texture, density), which may alter bone breakage on the macroscopic and microscopic scales [55]. This issue should not confound the results of this study, however, because damage inflicted to fresh bone will fossilize with those same morphological properties. Furthermore, tooth marks would not be produced on fossils and thus the comparison is not problematic.

Ideally, color observations can be used in bone modification analysis for assessing when the trace in question was generated relative to burial and/or fossilization. In many cases cross-cutting relationships with color heterogeneities provide insight into the relative timing for the origin of the trace, which then can be used to infer if the damage is recent, for instance from preparation or excavation [56, 57]. However, in many assemblages the outer and inner cortical-bone coloration is not different (e.g., bonebeds containing fossilized or unfossilized material that is mostly white). Under such a scenario, cross-cutting relationships with color variations do not occur [58]. Thus, the detailed morphology of the structure remains the most reliable criterion to infer if the trace in question is post-discovery or related to bioerosion.

The experimental air-scribe marks described herein were generated by a single device used by a single operator. Other chisel tips and/or types of scribes may introduce a host of slightly different morphologies. Further research is needed to elucidate the range of possible air scribe damage induced by variously shaped scribe tips and on fossils with various properties.

## Conclusions

There are morphological differences between the traces generated by air scribes, other commonly used excavation and preparation tools, and biostratinomic agents of bone-surface modification such as carnivores. Sequential, sub-millimeter scallops/craters are unique to the reciprocating impacts of the air scribe. Conversely, the presence of linearly continuous sub-features (i.e., internal grooves, shoulders, microstriations), which are often observed in marks produced by carnivore modification, trampling, or some tools, can be used to falsify the hypothesis that a trace in question was generated by an air scribe. A combination of the morphological criteria presented herein, cross-cutting relationships with other surficial features (e.g., diagenetic discoloration, weathering textures), the distribution of the modification, and an overall contextual framework for the assemblage is suggested for the interpretation of such traces. The ability to recognize or disprove air-scribe preparation damage will allow researchers to confidently proceed with interpreting past biological and sedimentological interactions with animal remains.
